# Can Psychological Contracts Decrease Opportunistic Behaviors?

**DOI:** 10.3389/fpsyg.2022.911389

**Published:** 2022-06-03

**Authors:** Leinan Zhang, Qingyan Zeng, Liu Yang, Yan Han, Yixin Xu

**Affiliations:** ^1^Department of Marketing, Business School, University of International Business and Economics, Beijing, China; ^2^Department of Management, Business School, University of International Business and Economics, Beijing, China; ^3^Department of Economics, School of Humanities and Social Sciences, Beijing Institute of Technology, Beijing, China

**Keywords:** behavioral psychology, psychological contracts, relationship quality, opportunism, dependence

## Abstract

Behavioral psychology is increasingly important in relationship marketing. As key factors of emotional interactions between buyer and seller, psychological contracts and opportunistic behaviors play a critical role in interorganizational relationships which are based on personal relationships of boundary spanners and top management. Most of the existing research mainly focus on positive performance of cooperation but ignoring the dark side of relationships. This study introduces the psychological contract into the exploration of why formal contracts cannot completely avoid opportunistic behaviors. It mainly investigates whether psychological contracts in relationships can reduce the occurrence of opportunistic behaviors. The results show that psychological contract has a significant positive effect on the relationship quality, and negatively affect opportunistic behavior through trust and commitment. The positive relationship between psychological contracts and relationship quality is moderated by dependence. This study enriches and expands the domestic and foreign research on psychological contracts and opportunistic behaviors in relationship marketing.

## Introduction

An increasing number of companies improve performance through relationship marketing. Due to all the interfirm relationship are individual relationship in nature, relationship marketing focus on more on behavioral psychology. Psychological contracts and opportunistic behaviors between buyer and seller provide new perspective of how to establish a long-term cooperative relationship, which will achieve greater success (Anderson and Weitz, 1992). This success is not only reflected in financial performance, but also conducive to improving innovation ability and value creation. Therefore, most of the existing relationship marketing studies are from a positive perspective, committed to exploring the activities and strategies that enable both parties to establish a positive relationship and create higher value. However, cooperation often has dark sides, including relationship conflict, opportunism, uncertainty and so on. These dark sides are inevitable, while the occurrence of such situations and behaviors can be reduced and controlled through effective management.

Furthermore, many studies have found that reducing the negative impact of the dark side of the relationship will have a greater impact on the success (Glavee-Geo et al., 2021; Rodrigues and Pinto-Ferreira, 2021). It is not enough to consider only the positive aspects of the relationship. We also need to explore how to restrain the dark side of relationship to help the cooperation between buyer and seller achieve greater success. In the past two decades, scholars have carried out extensive research on the dark side of buyer and seller relationship (Anderson and Jap, 2005; Mooi and Frambach, 2012), while compared with the research on the positive aspects of the relationship, the research on the dark side is far from sufficient. Among them, opportunism is considered to be the dark side of relationship, which increase the cost and reduce performance (Das and Rahman, 2010; Mellewigt et al., 2018). Reducing the opportunistic behavior can not only avoid the termination and destruction of cooperative relationship, but also reduce the transaction costs caused by unhealthy relationship, promote cooperation and create higher value.

The research on opportunistic behavior among buyer and seller has attracted an increasing attention from scholars. Many scholars have explored the essence and governance mechanism of opportunism (Wathne and Heide, 2000; Das and Rahman, 2010; Gould et al., 2016), but few scholars use psychological contract to explain opportunistic behavior in buyer and seller relationships. Part of the reason is that psychological contract was originally used to study the employer employee relationship within the organization.

The research in the field of psychological contract has mainly focused on the employment relationship, but many marketing scholars have shown that it is also important in relationship marketing (Roehling, 1997). Studies have found that psychological contract is related to trust and commitment (Kingshott, 2006; Hill et al., 2009; Herrera and Las Heras-Rosas, 2021), but it is a new perspective to explain opportunistic behavior. Therefore, based on social exchange theory and power dependence theory, this article aims to explore whether psychological contract can reduce the opportunistic behavior, and understand the mediator role of relationship quality and the moderator role of dependence. Relationship quality is divided into two dimensions, e.g., commitment and trust, which will provide deep insight of how psychological contract mitigate opportunistic behaviors (Alves et al., 2019; Qian et al., 2021).

## Literature Review

### Psychological Contract

The early psychological contract is based on the research in the fields of psychology and organizational behavior. Argyris (1960) first mentioned the concept of psychological work contract in his book. He described it as a silent agreement between the foreman and employees of the factory, but did not give a clear definition. Levinson et al. (1962) further analyzed and studied psychological contract, and was the first scholar to explicitly use the term “psychological contract” in the book. He defined it as the sum of mutual expectations between organizations and employees, and he believed that psychological contract was implicit to a great extent. In other words, psychological contract is different from written agreement. Psychological contract exists subjectively and only in individual thoughts (Rousseau, 1995).

Robinson et al. (1994) defined psychological contract as a belief held by an individual, that is, everyone is bound by a certain behavioral commitment related to the other party. Psychological contract can be considered as a strong psychological bond between the two parties (Anderson and Schalk, 1998). However, the most commonly accepted definition is proposed by Rousseau (1995), that is, personal belief shaped by the organization about the terms of individual and inter organization exchange agreement. In other words, employees believe that they have the obligation to act or perform in a certain way, and at the same time, they believe that the employer has certain obligations to them.

Some scholars use a single dimension to study and analyze psychological contract (Robinson et al., 1994; Kickul et al., 2002). Proposed a two-dimensional model of psychological contract, namely transactional psychological contract and relational psychological contract. Among them, transactional psychological contract pursues economic and external needs, and the boundary of responsibility is clear. Relational psychological contract pursues the satisfaction of social and emotional needs, and the boundary of responsibility is not clear. This dimension division is widely used by later scholars, and there are two main measurement methods: one is Rousseau's (2000) psychological contract inventory. The second is the psychological contract scale of Millward and Hopkins (1998). Based on above, the three-dimensional model adds the dimension of team members (Rousseau and Tijioriwala, 1996). The transaction dimension still emphasizes the exchange of specific, tangible and current interests. The relationship dimension here is defined as the mutual support and trust between the employer and the employee (between the team and members) and the commitment to long-term and open responsibilities. The team member dimension here specifically refers to that employees and organizations take responsibility for career development. In addition, Robinson (1995) also proposed a four-dimensional model. He believes that psychological contract includes four types: fluctuation type, stability type, relationship type and transaction type.

With the continuous development of research, the concept of psychological contract has also been used in other fields (Braganza et al., 2021). Anderson applied it to the study of teacher-student relationship. In recent years, more and more marketing scholars (Roehling, 1997; Pavlou and Gefen, 2005) believe that psychological contract may also have theoretical and management significance for relationship marketing. Roehling (1997) said that psychological contract also exists between consumers and enterprises. Pavlou and Gefen (2005) introduced psychological contract into the study of B2C relationship and explored the nature and role of violation of psychological contract (PCV) in online transactions between buyers and sellers. Similarly, Hill et al. (2009) also explored the violation of psychological contract. In the survey of suppliers of 110 large companies, they found that the violation of psychological contract will play an intermediary role in the trust of immoral behavior.

In addition, some studies have also confirmed the existence of psychological contract in B2B relationships (Blessley et al., 2018; Kingshott et al., 2020). Kingshott (2006) applied the psychological contract to the field of relationship marketing for the first time, exploring that the psychological contract between buyer and seller has a positive impact on the level of trust and commitment, and the stronger the psychological contract, the less likely it is to breach the contract. Generally speaking, there is still relatively little research on psychological contract in the field of relationship marketing. This article will explore the impact of psychological contract on opportunistic behavior to fill the gap in this field.

### Opportunistic Behavior

Existing studies have found that opportunism is the real dark force that has a negative impact on relationships (Crosno and Dahlstrom, 2008; Fang et al., 2011; Abosag et al., 2016). Due to the competitive objectives of buyer and seller, opportunism exists in almost every transaction to varying degrees (Luo, 2006). Existing literatures have found that the opportunistic behavior of buyer and seller is often the main reason for the sudden rupture of the cooperative relationship between the two sides (Das and Rahman, 2010). Therefore, understanding and paying attention to opportunistic behavior is very important for the progress and maintenance of buyer-seller cooperation.

Since 1980s, scholars at home and abroad have studied the opportunistic behavior for decades. This concept was originally defined by Williamson (1985) as the use of tricks for personal goal. Unlike the simple pursuit of self-interest, opportunistic behavior has fraudulent and intentional aspects (Wathne and Heide, 2000). Das and Rahman (2010) believe that opportunism is motivated by the desire to develop a relationship independently for personal gain or gain benefits, and tends to lead to short-term exploitation. Such behaviors include violating commitments, not sharing resources or facilities according to agreements, bluffing, lying, misleading, concealing, distorting, deceiving, misappropriating, stealing and other ways (Das and Rahman, 2010).

Opportunism is caused by many factors, which can be divided into three aspects: economy, relationship and time (Das and Rahman, 2010). Among, them, economy is an important factor widely recognized. When buyer and seller seek to obtain economic benefits or avoid economic losses, they will make opportunistic behavior against each other for their own interests, and the greater the economic amount involved, the greater the possibility of this behavior. The relationship factor refers to the tendency that an enterprise's perception of its partners will affect its opportunism (Ghoshal and Moran, 1996). Because buyer and seller do not feel guilty for their opportunistic behavior under the loose bilateral relationship (Das and Rahman, 2010). On the contrary, under the close communication and cooperation relationship, the frequent exchange of information will limit the possibility of opportunism itself. Time factor means that both parties have different expectations for the durability of the relationship. When the enterprise expects to maintain a long-term relationship with the other party, it will inhibit its opportunistic behavior. On the contrary, time pressure will also lead to opportunistic behavior. In addition, Gould et al. (2016) considered that transaction specific investments, behavioral uncertainty, environmental uncertainty and frequency of exchanges would lead to opportunistic behavior.

Opportunism has many forms. Williamson (1985) divided it into positive and negative aspects according to the purpose of behavior. Among them, the positive aspect refers to opportunistic behaviors such as violating commitments and concealing information in order to obtain their own interests. The negative aspect refers to the enterprise in order to avoid its responsibilities. In addition, Luo (2006) divided opportunism into two forms: weak opportunism and strong opportunism, which is also one of the most widely used classification forms in later scholars' research. Among them, strong opportunism involves violations of contract norms, which are clearly incorporated into the main body of the contract and various supplementary clauses signed in the later stage. Weak opportunism involves violations of relationship norms, which are not stated in the contract, but are rooted in the consensus of all members in a specific relationship, thereby harming the interests of the other party.

Opportunistic behavior itself will have a negative impact on the cooperative relationship between the two sides and greatly reduce the cooperation enthusiasm of the two sides. Although opportunistic behavior will increase short-term returns, in the long run, it is not conducive to the value creation of both parties. On the one hand, opportunistic behavior will reduce the innovation intention. The higher the level of opportunism of its partners, the fewer opportunities for buyers to be encouraged to innovate (Mooi and Frambach, 2012). On the other hand, opportunism will reduce the tacit understanding of cooperation between the two sides of the transaction, and the common income largely depends on the degree of synergy between the two sides of the transaction (Dyer, 1997). In addition, because opportunistic behavior is difficult to detect and verify, buyer and seller that perceive this threat will face the need for further screening, negotiation and supervision of partners, resulting in increased information costs (Hennart, 1988).

### Relationship Quality

Relationship quality refers to the overall nature of the relationship between the two parties (Hennig-Thurau et al., 2002; Liu et al., 2010). Buyer and seller with high relationship quality trust each other and have confidence in future performance (Crosby et al., 1990). Although existing studies have not reached a consensus on the dimensions of relationship quality, for example, Kumar et al. (1995) believe that relationship quality includes conflict, trust and commitment. Gao et al. (2009) believe that relationship quality also includes coordination and response. Generally speaking, trust and commitment are the two most important variables to describe and measure relationship quality (Anderson and Weitz, 1992; Chua and Morris, 2008; Fang et al., 2008, 2011). Trust and commitment are also the basis and important factors for establishing, developing and maintaining successful buyer-seller relationships (Morgan and Hunt, 1994; Berry, 1995).

Trust is an important element to establish and maintain long-term relationships. It has been widely studied by scholars in different fields such as psychology, sociology, management and economics. It has also been defined from different angles. Geyskens et al. (1996) defined trust as trust in the honesty and ability of others. Moorman et al. (1993) believe that trust is the confidence to rely on the will of its trading partners. Although expressed differently, both definitions emphasize confidence and reliability (Garbarino and Johnson, 1999). In addition, willingness to take risks is considered to be one of the characteristics of trust (Johnson-George and Swap, 1982). Gambetta (1988) believes that trust is fragile. Based on this, Mayer et al. (1995) believe that trust is that one party is willing to bear the attack and harm that may be caused by the other party's behavior based on the expectation that the other party will perform specific actions important to itself, without considering whether it can supervise or control the other party.

In order to further explain trust, Mayer et al. (1995) explained trust from three dimensions: ability, kindness and integrity. Kumar et al. (1995) also divided the dimensions of trust in their own research, believing that trust includes kindness and honesty. Similarly, Geyskens et al. (1996) divided trust into honesty, kindness and ability. Among them, honesty describes the situation that both parties share information without concealing or deliberately increasing false information. Capability means that partners have sufficient professional knowledge and capability to provide stable product quality and quantity. Kindness refers to considering the interests of partners and being willing to take actions to protect the interests of partners. In addition, some scholars divide trust from two dimensions: cognitive based trust and affect based trust (McAllister, 1995; Johnson and Grayson, 2005; Chua and Morris, 2008). This article mainly explores the trust relationship from the perspectives of honesty, kindness and ability.

Commitment is also an important factor for the maintenance and long-term development of bilateral relationship. Commitment was first used in the field of social exchange (Blau, 1964) and is considered as a variable to distinguish social exchange from economic exchange (Cook and Emerson, 1978). Subsequent scholars extended it to the study of relationship marketing. It is considered that commitment refers to the willingness of trading partners to maintain a long-term relationship, and the commitment party believes that it is essential to make the best efforts to maintain the sustainable development of the relationship with the other party (Morgan and Hunt, 1994). Anderson and Weitz (1992) defined commitment from the perspective of the buyer, specifically referring to the buyer's desire to develop a stable relationship, willingness to make short-term sacrifices to maintain this relationship, and confidence in the stability of the relationship with suppliers. It can be seen that commitment includes both attitude and behavior, and the definition focuses more on the description of behavior (Ashnai et al., 2016).

In order to further explain and measure commitment, Gundlach et al. (1995) divided it into three dimensions: tool dimension, attitude dimension and time dimension. From the perspective of instrumental dimension, commitment is a calculable behavior. Attitude dimension refers to emotional commitment, that is, emotional dependence on organizational goals and values. Time dimension refers to long-term commitment, that is, the relationship can exist stably in the future.

Commitment is an important part of a successful long-term relationship. Like trust, it will also directly affect the cooperative behavior, thus affecting whether relationship marketing can succeed. Partners can benefit from high commitment (Fang et al., 2011). Xiaorong et al. (2016) showed that a high degree of commitment will improve the performance level of bilateral cooperation. Stanko et al. (2007) found that commitment can promote favorable buyer purchase behavior, that is, buyers make stable, frequent and large purchases. At the same time, Lam et al. (2004) believe that commitment is related to the conversion cost, and a high degree of commitment will increase the conversion cost. The high conversion cost will reduce the possibility of customers changing their partners, which means that the relationship between the two sides of the transaction is more stable.

### Dependence

Dependence was first defined as the degree of influence between organizations or individuals. In later studies, some scholars defined inter-organizational dependence as the importance and irreplaceable degree of resources provided by partners based on the viewpoint of resource dependence theory. The higher the scarcity or importance of resources, the higher the degree of dependence. Reflected in the relationship marketing, this dependence comes from the extent to which the buyer needs to specify its partners, that is, whether its partners provide the buyer with vital interests or whether there are other replaceable resources in the market (Tellefsen and Thomas, 2005). As the importance of benefits increases and the availability of alternatives decreases, so does dependence (Emerson, 1962; Luo et al., 2011). In addition, there are many other forms of dependence. The substitutability of existing partners is one of them (Heide and John, 1988), that is, when an enterprise cannot find another similar enterprise to replace the existing partner, it will rely on the partner.

Generally speaking, there are three main aspects of dependence: first, the importance of resources, that is, buyer and seller need this resource very much. Second, the other party has and can control the resources freely. Third, there are few or almost no potential alternative resources or alternative partners (Heide and John, 1988). For the dimension of dependence, there is no unified conclusion in the academic circles. In some studies, related to marketing and supply chain, it is considered that the directionality (positive/negative) of the demand that depends on both parties to maintain the exchange relationship is way of measuring dimension of dependence. Among them, positive motivation means that it will bring irreplaceable relationship benefits to itself, including sales benefits (Heide and John, 1988), other resources, the importance of partners, etc. Negative motivation refers to avoiding or reducing the conversion cost caused by changing partners (Anderson and Narus, 1990). In addition, Scheer et al. (2010) divided dependence into benefit based and cost based in their research on dependence between customers and buyer and seller. Among them, benefit-based dependence refers to the demand for maintaining relationship due to irreplaceable net income. Cost based dependency refers to the need to maintain the relationship due to the huge cost of the end of the relationship.

Dependence plays an important role in relationship marketing, so more and more scholars have carried out extensive research on dependence among channel members. Some scholars believe that Inter Organizational dependence can enhance channel relationship (Ganesan, 1994; Tellefsen and Thomas, 2005). Because when the dependence increases, that is, the value of income increases and the number of alternatives decreases, buyers are more inclined to maintain this relationship and increase their commitment to this relationship (Syed and Andaleeb, 2001). At the same time, when dependence increases, the exchange of knowledge, capabilities and resources between its partners and buyers becomes closer, which is more likely to promote technological innovation (Sengun et al., 2014). In addition, increasing the degree of dependence between buyer and seller contributes to the construction of internal relationship, which has a positive impact on performance (Özen et al., 2016).

This article will fill the gaps in the above aspects and explore the role of psychological contract between buyer and seller on opportunistic behavior. At the same time, because psychological contract is implicit and subjective, it cannot be directly perceived by the other party and affect the other party's behavior. Therefore, this article selects relationship quality as the intermediate variable, and trust and commitment as the two variables of relationship quality. On this basis, explore the moderator role of dependence in this mechanism.

## Research Model and Hypothesis Development

### Research Model

**Figure 1:**
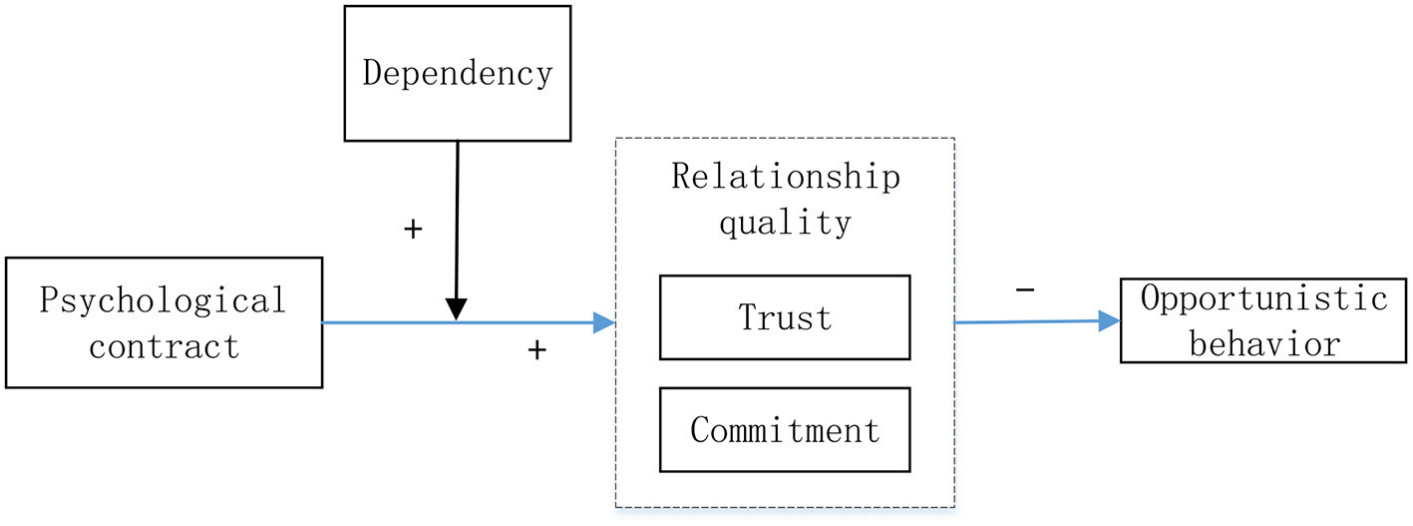
Research model.

Opportunistic behavior is the dark force in the enterprise relationship marketing. Serious opportunistic behavior will even lead to the direct rupture of the cooperative relationship between the two sides. This article mainly explores how psychological contract reduces the opportunistic behavior of partners in channel relationship and the moderator role of dependence. In this model, the independent variable is psychological contract, the dependent variable is the opportunistic behavior of buyer and seller, and the relationship quality is the mediator variable. Partner dependence is the moderating variable, and their changes will affect the effect of psychological contract on relationship quality. The conceptual model is shown in Figure 1.

### Hypothesis Development

#### Psychological Contract and Relationship Quality

Rousseau (1995) defined psychological contract as individuals believe in the common obligations between themselves and the other party. In other words, psychological contract includes one party's understanding of himself and the other party's relationship obligations (Anderson and Schalk, 1998). Specific to the relationship marketing, that is, what the enterprise expects its partners to improve for him, and what obligations he needs to provide for his partners. Psychological contract is an important tool to explain the relationship between buyer and seller, and the psychological contract is dynamic and will change with the development of the relationship between the two parties and continuous communication (De Meuse et al., 2001). Trust and commitment in relationship quality are crucial factors for the maintenance and development of bilateral relationship. Therefore, this article believes that psychological contract will have a positive effect on relationship quality.

#### Psychological Contract and Trust

When the psychological contract is stronger, the enterprise has a clearer understanding and positioning of its partnership obligations, and the expectation of the other party to fulfill its relationship obligations will increase (Dwyer and Paul, 1987). And psychological contract is different from other forms of contract, which is based on their own perception (Kingshott, 2006). This article holds that psychological contract will have a positive effect on trust for the following reasons:

Firstly, when an enterprise perceives its partners' positive attitude toward relationship maintenance, it will believe that its partners will take reliable and honest behavior to meet organizational needs (Anderson and Narus, 1990), that is, the enterprise will have strong trust in the honesty of its partners. Secondly, when the enterprise perceives that the enterprise partner has a high sense of responsibility and commitment to the relationship and a high level of cooperation, it will think that the partner will pay attention to the interests of the company and strive for common interests, that is, the enterprise's benevolent trust in its partners. Moreover, when an enterprise believes that its partner can provide it with the required tools, equipment, information and other resources, it will greatly recognize the professionalism and ability of the partner, that is, the enterprise's trust in the ability of its partner.

Based on the above aspects, when the enterprise has a clear positioning for the commitments of its partners and believes that these commitments are of great help to itself and the development of cooperative relationship, it will be more willing to maintain a longer-term relationship with its partners, greatly reducing the sense of insecurity and uncertainty of the relationship. Therefore, under a strong psychological contract, buyer and seller trust in their partners will be enhanced.

#### Psychological Contract and Commitment

Commitment is also an important aspect of relationship quality, which is defined as one party believes that the other party is important enough to make the best effort to maintain the relationship (Anderson and Weitz, 1992; Morgan and Hunt, 1994; Herrera and Las Heras-Rosas, 2021). This article holds that psychological contract will also have a positive effect on commitment. The specific reasons are as follows:

First of all, when the psychological contract is strong, it means that buyer and seller are very clearly aware of their commitments and obligations, and think that such promises will bind them to some form of future relationship. Therefore, buyer and seller will try their best to fulfill their obligations to meet each other's expectations and needs, so that buyer and seller can obtain greater benefits from good cooperative relationship. Then, buyer and seller will be more patient with the partner and willing to make short-term sacrifices to maintain the relationship, which is reflected in the enhanced commitment of the buyer and seller to its partner.

Secondly, the clear positioning of the buyer and seller for its partners to perform their duties and the perception of the commitments made by its partners will make the buyer and seller more confident in the future development of the relationship, and then more willing to invest more resources in the relationship and strengthen the contact with the partner, which is also reflected in the enhanced commitment of the buyer and seller to its partners. Therefore, under a strong psychological contract, the commitment of buyer and seller to their partners will be enhanced.

On the contrary, when one party perceives that the psychological contract has been broken or violated, it will think that the other party's words and deeds are inconsistent, so it loses confidence in the promised return of the current payment in the future, resulting in reduced trust in the other party and reduced its motivation to pay for the relationship, so it will no longer give a high degree of commitment. Therefore, psychological contract will positively affect relationship quality, that is, the stronger the psychological contract, the stronger the trust and commitment.

To sum up, this article puts forward the following hypotheses:

H1: psychological contract is positively correlated with relationship quality.
H1a: psychological contract is positively correlated with trust.
H1b: psychological contract is positively correlated with commitment.

#### Relationship Quality and Opportunistic Behavior

Opportunism is the dark side of the cooperative relationship between buyer and seller, often for private interests rather than common interests among buyer and seller. The occurrence of opportunistic behavior may destroy or even lead to the termination of cooperative relationship. This article argues that relationship quality will negatively affect the opportunistic behavior of partners. Because closer partnerships focus on the longer-term future rather than short-term interests. Trust and commitment are important factors to measure relationship quality (Fang et al., 2011).

#### Trust and Opportunistic Behavior

Trust is considered to be a belief in the honesty, kindness and ability of its partners (Kumar et al., 1995; Mayer et al., 1995; Geyskens et al., 1996). This article holds that high trust in partners will reduce their opportunistic behavior. The main reasons are as follows:

First of all, believing in the honesty and kindness of partners means believing that partners will adopt honest behavior and attitude in the process of cooperation, consider our interests and will not do anything against their commitments and obligations. Therefore, buyer and seller will be more assured to improve their real information. The improvement of transaction transparency reduces the counterparty's perception of risk and uncertainty (Shao et al., 2020). In addition, trust also means that buyer and seller are willing to take risks and put themselves in a weak position vulnerable to each other (Gambetta, 1988). This will also reduce their partners' perception of uncertainty. The reduction of uncertainty will reduce the opportunistic behavior of its partners to avoid losses and risks.

Secondly, trust the ability of partners, which means that buyer and seller believe that their partners have high professional knowledge and skills and can provide them with great help. In this case, buyer and seller will more cooperate and communicate with their partners, let their partners participate in their important decisions, and give their partners a very important role in this partnership. This trust forms a “moral constraint” on its partners, so that its partners have to focus on common interests and long-term interests, so as to reduce or avoid the opportunistic behavior of its partners.

Moreover, when buyer and seller have high trust in their partners, they will be willing to invest more resources and energy in this partnership and maintain more patience with their partners. This series of behaviors express the willingness and sincerity of the enterprise for long-term cooperation, so that its partners will consider more long-term plans rather than short-term interests, and then reduce the occurrence of opportunistic behavior.

#### Commitment and Opportunistic Behavior

At the same time, this article holds that commitment to partners will also reduce their opportunistic behavior. The main reasons are as follows:

On the one hand, commitment is manifested in attitude and desire to develop stable relationships (Anderson and Weitz, 1992), which refers to the lasting desire to maintain precious relationships (Fang et al., 2011). Therefore, when an enterprise has a high commitment to its partners, it means that the enterprise hopes to maintain a long-term cooperative relationship with its partners and believes that its partners are very important to it. This long-term cooperation intention and attitude will promote buyer and seller to communicate more closely with their partners, so as to reduce the uncertainty about the future development of bilateral relationship caused by information asymmetry and enhance the sense of security of their partners. It is this increased sense of security and the expectation of long-term cooperation in the future that avoid or reduce the opportunistic behavior of its partners due to risk aversion.

On the other hand, when the commitment is higher, more resources and energy will be invested in maintaining the relationship. With the input of more and more resources, the relationship between the two sides continues to strengthen, involving more and more common interests. Bad cooperation will reduce the input-output ratio. Opportunistic behavior is a selfish behavior that only pays attention to its own interests. It is a behavior that obtains its own interests at the expense of the interests of others (Williamson, 1985). When their partners find that they are highly related to the interests of our enterprise, they will have to give up the behavior of harming each other's interests in order to create greater common value, so as to reduce or avoid the opportunistic behavior of their partners.

To sum up, this article puts forward the following hypotheses:

H2: relationship quality is negatively correlated with opportunistic behavior.
H2a: trust is negatively correlated with opportunistic behavior.
H2b: commitment is negatively correlated with opportunistic behavior.

#### The Mediating Role of Relationship Quality

Psychological contract is implicit and only exists in individual thoughts and consciousness (Rousseau, 1995). Therefore, the positioning and perception of their own obligations and the obligations of the other party cannot be directly perceived by others. Moreover, the psychological contract is subjective. Both parties have their own cognition of their own and each other's obligations, and the cognition of both parties can be inconsistent and not recognized by the other party. The psychological contract does not emphasize that both parties reach an agreement on this cognition. Therefore, the enterprise's psychological contract cannot directly affect the behavior of its partners, can only affect their own behavior. When one party has a strong psychological contract, it will be shown through trust and commitment. Therefore, this article holds that relationship quality plays an intermediary role in the influence of psychological contract on opportunistic behavior.

Moreover, the psychological contract within the organization defines what employees think the organization has promised them and what they think they have the obligation to contribute in return. Therefore, it can be understood that psychological contract includes two parts: my positioning of my own commitments and obligations and my views and beliefs on the obligations and commitments of others. If one party perceives that the other party has not fulfilled its implicit or explicit commitment, even if it has fulfilled its commitment, the psychological contract will be destroyed (Alcover et al., 2017).

In the relationship marketing, when the psychological contract is strong, it not only means that the enterprise is clear about its own commitments and obligations, but also means that the enterprise has the belief and perception of its partners to fulfill their commitments. In other words, under the strong psychological contract, the buyer and seller perceives that its partners have made efforts to meet their needs and interests, so it believes that its partners will fulfill their commitments and obligations in the future. This will make the communication between the enterprise and its partners closer, and this trust is also reflected in the sincerity and honesty of the enterprise to its partners in cooperation, which increases the “sense of security” of its partners in this relationship, and then reduces or avoids their opportunistic behavior to avoid risks. Therefore, trust plays an intermediary role in the influence of psychological contract on opportunistic behavior.

At the same time, buyer and seller are more willing to invest more resources and energy in this relationship because they perceive that their efforts have received due response and their belief that their partners will perform their future responsibilities, that is, buyer and seller have shown strong commitment to their partners. With the continuous increase of input resources, it also increases the conversion cost for buyer and seller to convert their partners, and enables buyer and seller to maintain a healthy and close cooperative relationship with their partners in order to further maintain the invested resources, so as to obtain the promotion of common value. This concept and method of long-term cooperation oriented relationship management will promote partners to participate more in decision-making, and also focus on the long-term future, so as to avoid the problem that they only focus on short-term interests without considering whether the cooperation relationship will be maintained for a long time Opportunistic behavior. Therefore, commitment plays an intermediary role in the influence of psychological contract on opportunistic behavior.

To sum up, this article puts forward the following hypotheses:

H3: relationship quality plays a mediating role in the impact of psychological contract on opportunistic behavior.
H3a: trust plays a mediating role in the influence of psychological contract on opportunistic behavior.
H3b: commitment plays a mediating role in the influence of psychological contract on opportunistic behavior.

#### The Moderating Effect of Dependence

Dependence is very important in relationship marketing management and development. In essence, marketing channels are composed of interdependent organizations. Based on the theory of power dependence, when one party is difficult (or unable) to find a substitute, it will form dependence on the other party. In enterprise relationship, dependence specifically refers to the need for the company to maintain a relationship with specific partners to achieve its goals. This article holds that the degree of dependence will positively regulate the impact of psychological contract on relationship quality. The main reasons are as follows:

First of all, the higher the enterprise's dependence on its partners, it means that the partners have important resources required by the enterprise or have a more important impact on the enterprise's profits or innovation. In this case, the enterprise will think that its partners can provide more help and benefits to create higher value, which will be reflected in the psychological contract, so that the enterprise has greater confidence in the development of future relationship, as well as more confidence that its partners will fulfill their commitments and future responsibilities. Furthermore, the enterprise will let its partners more participate in important decisions, believe that the other party will pay attention to our interests and provide us with help and support at any time, that is, the enhancement of trust in partners. Therefore, when the degree of dependence is higher, the impact of psychological contract on trust will be strengthened.

Secondly, when an enterprise is more dependent on its partners, it is difficult for the enterprise to find other partners. It often needs to pay more costs to terminate this relationship and change supply channels, resulting in higher economic losses. Therefore, in this case, it is not a wise choice for them to end this relationship and seek new partners. Buyer and seller are more inclined to maintain stable relationship, seek the long-term development of cooperative relationship, and obtain higher interests by maintaining close relationship. This awareness of long-term cooperation makes buyer and seller more clearly aware of their future obligations and responsibilities, have a stronger sense of responsibility, and believe that they need to make efforts to maintain the enterprise relationship between the two sides. Thus, buyer and seller will invest more resources and energy in this relationship to achieve long-term interests. Commitment is expressed as a desire to develop a stable relationship and a willingness to make short-term sacrifices to maintain this relationship (Tse et al., 2019). Therefore, the higher the degree of dependence, the stronger the impact of psychological contract on commitment.

On the contrary, when buyer and seller rely less on their partners, it means that buyer and seller can easily find other partners with similar price and quality, and the conversion cost is low. Therefore, the lower the degree of dependence, the less attention buyer and seller pay to this relationship and the less “sense of responsibility” for maintaining the relationship and promoting value creation. In this case, buyer and seller will think that the future development of this relationship is uncertain, and naturally they will not invest more energy and resources in this relationship. Therefore, the lower the degree of dependence, the weaker the impact of psychological contract on trust and commitment in relationship quality.

To sum up, this article puts forward the following hypotheses:

H4: the degree of dependence will positively moderate the impact of psychological contract on relationship quality.
H4a: when the degree of dependence increases, the positive correlation between psychological contract and trust will increase.
H4b: when the degree of dependence increases, the positive correlation between psychological contract and commitment will increase.

## Methodology

This study obtains relevant data by questionnaires, and then use the method of statistical analysis to test the conceptual model and hypothesis. In terms of the setting of questionnaire items, it is divided into basic characteristics and research related items. Among them, the basic characteristics of the enterprise, establishment time, number of employees and annual sales are the control variables of the research. The research related items include psychological contract, relationship quality (trust and commitment), opportunistic behavior and dependence.

Because there are many studies on psychological contract, relationship quality, opportunistic behavior and dependence in foreign related fields, most of the indicators have been empirically tested and have high reliability and effectiveness, this article will design and complete the structural questionnaire of this study according to the research in related fields and using the mature scale of relevant literature for reference. In order to avoid the common method deviation caused by the data coming from the perceived information of the same respondent at a certain time, this study divided the questionnaire topics into two sets of questionnaires A and B. Among them, questionnaire A is related to psychological contract, relationship quality (trust and commitment) and dependence, and questionnaire B is related to opportunistic behavior. After the preliminary questionnaire is formed through many tests and amendments, a small-scale pre-test is carried out to further rationalize the problems in the first draft, so as to ensure the reliability of the scale.

### Measurement

The purpose of this article is to explore the effect of psychological contract on the occurrence of opportunistic behavior among buyer and seller, involving five variables: psychological contract, trust, commitment, opportunistic behavior and dependence. In order to ensure the accuracy of variable measurement and the reliability of data collection, the existing mature scales are used for all variables in this study, but they are slightly modified according to the research content and purpose of this article. And use Likert's seven-point scale to measure variables in this study.

#### Psychological Contract

The concept of psychological contract has been regarded as an important tool for understanding the employment relationship, that is, a measure of the relationship between the organization and employees. In the field of relationship marketing between buyer and seller, Kingshott (2006) investigated and studied the psychological contract between buyer and seller for the first time on the basis of Rousseau and Tijioriwala (1996). The measurement of psychological contract in this study adopts the four factor scale proposed by Kingshott (2006) on the basis of Rousseau and Tijioriwala (1996), which include 22 items.

#### Relationship Quality

Relationship quality refers to the overall nature of the relationship between the two parties. There are different discussions on the dimensions of relationship quality. This article selects two widely recognized dimensions, trust and commitment, to measure relationship quality. Among them, the measurement of trust adopts and combines the scale of Kumar et al. (1995) and Mayer et al. (1995), which will be measured from the three dimensions of honesty, kindness and ability. Each dimension is set with 3 items, a total of 9 items. In addition, the four items of commitment were selected from the scale of Anderson and Weitz (1992).

#### Opportunistic Behavior

The essence of opportunistic behavior is the concealment of the other party's deception-oriented behavior and the violation of the other party's requirements or expectations. It is mainly manifested in two aspects: concealing information and failing to fulfill commitments. Its main purpose is to seek its own interests and act based on self-interest. The opportunistic measurement in this article mainly adopts the maturity scale in the published articles of Wuyts and Geyskens (2005) and Huo et al. (2015), and sets up five items from the above two aspects.

#### Dependence

Dependency refers to the need to maintain a relationship with another party to achieve the ultimate goal. Therefore, when one party's expected goal needs to be achieved by the other party's specific behavior, it depends on the other party according to the power dependence theory. At the same time, according to the resource dependence theory, dependence arises from the fact that partners can provide important or irreplaceable resources. Based on the above two theories, this article mainly adopts the dependence correlation scale of Kim and Hsieh (2003), including four items.

### Data Collection

This study explores the impact of psychological contract in relationship marketing on enterprise opportunistic behavior. Therefore, buyer and seller with inter enterprise transactions are selected as the specific research object, and two questionnaires are distributed to two relevant management employees participating in the enterprise cooperation relationship, and both parties are informed that they need to answer relevant questions to the same enterprise customers, so as to avoid the common method deviation caused by the perceived information of the same respondent at a certain time. A total of 300 questionnaires were distributed in this study, and 151 questionnaires were actually recovered, with a recovery rate of 50.33%.

There were 127 valid questionnaires, and the effective rate was 84.1%.

### Hypothesis Test

Reliability analysis is mainly used to test the consistency of the measurement results and the reliability of the results. In this article, Cronbach's alpha reliability coefficient method is used to analyze the data by IBM SPSS 24.0 software. The Cronbach's α values of each variable dimension are >0.7, which shows that the reliability of each dimension of this study has reached the standard, with high consistency and reliability.

Pearson correlation coefficient is used to analyze the correlation degree and linear relationship direction of the relationship between two variables. the specific data results are shown in Table 1.

**Table 1:** Correlation analysis results.

**Variable**	**Mean**	**SD**	**PC**				**TR**			**CR**	**DP**	**OPP**
			**GFFD**	**RB**	**RC**	**IRC**	**TH**	**TB**	**TA**			
GFFD	5.972	1.077	1									
RB	5.265	1.226	0.715^**^	1								
RC	5.797	0.865	0.656^**^	0.672^**^	1							
IRC	5.633	1.073	0.814^**^	0.809^**^	0.669^**^	1						
TH	5.478	0.915	0.642^**^	0.794^**^	0.563^**^	0.751^**^	1					
TB	5.535	1.200	0.728^**^	0.798^**^	0.578^**^	0.837^**^	0.734^**^	1				
TA	5.727	0.824	0.686^**^	0.716^**^	0.791^**^	0.680^**^	0.572^**^	0.746^**^	1			
CM	5.545	1.016	0.639^**^	0.762^**^	0.670^**^	0.741^**^	0.706^**^	0.716^**^	0.730^**^	1		
DP	5.199	1.163	0.656^**^	0.776^**^	0.539^**^	0.762^**^	0.786^**^	0.699^**^	0.560^**^	0.815^**^	1	
OPP	2.249	1.045	−0.630^**^	−0.731^**^	−0.676^**^	−0.772^**^	−0.739^**^	−0.745^**^	−0.666^**^	−0.768^**^	−0.701^**^	1

** *There was significant correlation at the level of 0.01 (bilateral)*.

*PC, psychological contract; Tr, trust; Cm: commitment; Opp, opportunistic behavior; DP, dependency*.

In the regression model of psychological contract and relationship quality, the independent variable is psychological contract, the dependent variable is relationship quality (trust and commitment), and the establishment time, number of employees and annual sales are the control variables. Among them, the data processing of trust TR and psychological contract PC adopts the weighted average method, that is, TR is the average value of th, TB and Ta, and PC is the average value of gffd, Rb, RC and IRC. This method is used for the following regression analysis and test data processing. The effects of psychological contract on the two dimensions of relationship quality are shown in Table 2.

**Table 2:** Regression analysis results of psychological contract and relationship quality.

	**Trust**		**Commitment**	
	***β***	**Significance**	***β***	**Significance**
Establishment time	0.067	0.185	0.150	0.037^*^
Number of employees	−0.101	0.104	−0.164	0.064
Annual sales	0.076	0.188	0.053	0.512
Psychological contract	0.903	0.000^***^	0.786	0.000^***^
*R* square	0.823		0.643	
Adjusted *R* square	0.817		0.631	
F	141.968		54.910	
DW	2.120		1.810	

*** *Means the significance level is 0.001*.

* *Means the significance level is 0.05*.

The data results show that the regression coefficient of psychological contract and trust is 0.903 and the significance value is 0.000, which shows that psychological contract has a significant positive effect on trust, so hypothesis 1a is verified. Moreover, the adjusted R square is 0.817, indicating that the overall fitting degree of the model is good. The F value of the model is 141.968. The DW value is 2.120, which is near 2, indicating that there is no strong autocorrelation between the variables of the model.

At the same time, the regression coefficient of psychological contract and commitment is 0.786 and the significance value is 0.000, which shows that psychological contract also has a significant positive effect on commitment, so hypothesis 1b is also supported. In addition, the adjusted R square is 0.631, indicating that the overall fitting degree of the model is good. The F value of the model is 54.910. The DW value is 1.810, close to 2, so the residual sequence has no autocorrelation.

In the regression model between relationship quality and opportunistic behavior, the independent variable relationship quality (trust and commitment), the dependent variable is opportunistic behavior, and the establishment time, number of employees and annual sales are the control variables. The effect on opportunistic behavior is shown in Table 3.

**Table 3:** Regression analysis results of relationship quality and opportunistic behavior.

	**B**	***β***	**Significance**
Establishment time	−0.022	−0.019	0.774
Number of employees	−0.022	−0.037	0.652
Annual sales	0.090	0.114	0.133
Trust	−0.642	−0.538	0.000^***^
promise	−0.344	−0.334	0.000^***^
*R* square		0.698	
Adjusted *R* square		0.686	
F		56.030	
DW		1.582	

*** *Means the significance level is 0.001*.

^**^Means the significance level is 0.01.

^*^Means the significance level is 0.05.

The data results show that the regression coefficient between trust and opportunistic behavior is −0.538 and the significance value is 0.000, which shows that trust has a significant negative effect on opportunistic behavior, so hypothesis 2a is verified. The regression coefficient between commitment and opportunistic behavior is −0.334 and the significance value is 0.000, which indicates that commitment also has a significant negative effect on opportunistic behavior, so hypothesis 2b is supported. Moreover, the adjusted R square is 0.686, indicating that the overall fitting degree of the model is good. The F value of the model is 56.030. The value is close to DW 581.22, which shows that there is no strong autocorrelation between the variables of the model.

This study tests the mediating effect of relationship quality in psychological contract and opportunistic behavior, and controls the establishment time, number of employees and annual sales. The mediation effect is listed in Table 4.

**Table 4:** Mediation effect results of trust.

	**Trust**		**Opportunistic behavior**		**Opportunistic behavior**		**Opportunistic behavior**	
	***β***	***t***	***β***	***t***	***β***	***t***	***β***	***t***
Establishment time	0.067	1.332	−0.053	−0.754	−0.104	−1.437	−0.070	−1.019
Number of employees	−0.101	−1.639	−0.007	−0.076	0.069	0.771	0.017	0.198
Annual sales	0.0760.076	1.323	0.116	1.460	0.054	0.658	0.093	1.193
Psychological contract	0.903	23.592^***^			−0.782	−14.148^***^	−0.318	−2.602^***^
Trust			−0.803	−15.076^***^			−0.514	−4.185^***^
*R* square	0.823		0.659		0.631		0.677	
F	141.968		58.993		52.048		50.780	

*** *Means the significance level is 0.001*.

*All coefficients are standardized coefficients*.

It can be seen from the data in the above table that psychological contract is significantly correlated with trust at the level of 0.001, R square is 0.823 and F is 141.968, which shows that psychological contract has a significant positive effect on trust. At the same time, trust is significantly correlated with opportunistic behavior at the level of 0.001, R square is 0.677, F is 50.780, which shows that trust has a significant negative effect on opportunistic behavior. To sum up, trust plays a significant mediating effect in the impact of psychological contract on opportunistic behavior.

Similarly, it can be seen from the data in the above table that psychological contract is significantly correlated with commitment at the level of 0.001, R square is 0.643 and F is 54.910, indicating that psychological contract has a significant positive effect on commitment. At the same time, commitment is significantly correlated with opportunistic behavior at the level of 0.001, R square is 0.595, F is 44.842, which shows that commitment has a significant negative effect on opportunistic behavior. To sum up, commitment plays a significant mediating effect in the impact of psychological contract on opportunistic behavior. Please refer it to Table 5.

**Table 5:** Mediation effect results of commitment.

	**Commitment**		**Opportunistic behavior**		**Opportunistic behavior**		**Opportunistic behavior**	
	***β***	***t***	***β***	***t***	***β***	***t***	***β***	***t***
Establishment time	0.150	2.111	0.005	0.062	−0.104	−1.437		−0.689
Number of employees	−0.164	−1.868	−0.038	−0.404	0.069	0.771	0.007	0.082
Annual sales	0.053	0.658	0.098	1.134	0.054	0.658	0.075	0.965
Psychological contract	0.786	14.452^***^			−0.782	−14.148^***^	−0.486	−5.722^***^
Commitment			−0.768	−13.117^***^			−0.377	−4.395^***^
R square	0.643		0.595		0.631		0.681	
F	54.910		44.842		52.048		51.754	

*** *Means the significance level is 0.001*.

^**^Means the significance level is 0.01.

^*^Means the significance level is 0.05.

*All coefficients are standardized coefficients*.

#### Moderating Effects of Dependent

This article uses linear regression to test the moderating effect of dependence on psychological contract and relationship quality, and controls the establishment time, number of employees and annual sales. The dependent regulatory effects are listed in Table 6.

**Table 6:** Regression analysis results of dependent regulatory effects.

	**Trust**		**Commitment**	
	***β***	***t***	***β***	***t***
Establishment time	0.056	1.152	0.116	2.141^*^
Number of employees	−0.126	−2.101^*^	−0.243	−3.602^***^
Annual sales	0.099	1.775	0.125	2.012^*^
Psychological contract	0.817	13.119^***^	0.537	7.692^***^
Rely on	0.169	2.909^**^	0.504	7.748^***^
Rely on × Psychological contract	0.089	2.042^*^	0.283	5.756^***^
*R* square	0.832		0.789	
F	104.990		79.466	

*** *Means the significance level is 0.001*.

** *Means the significance level is 0.01*.

* *Means the significance level is 0.05*.

It can be seen from the data in the table that the significance of the interaction between dependence and psychological contract and trust is <0.05, which means that dependence plays a moderator role in the impact of psychological contract on trust. Hypothesis 5A is verified. In addition, R square is 0.832 and F value is 104.990. At the same time, the significance of the interaction between dependence and psychological contract and commitment is <0.001, so there is a significant positive effect, and the R square is 0.789 and the F value is 79.466. Therefore, dependence has a positive moderator role in psychological contract and commitment, and hypothesis 5b is supported.

## Conclusions and Prospect

### Research Conclusions

Based on the theory of power dependence and social exchange, this article explores the mechanism of psychological contract on opportunistic behavior under relationship marketing, the intermediary role of relationship quality in this role, and the moderator role of dependence in psychological contract and relationship quality. The main conclusions are as follows:

Firstly, psychological contract has a significant positive impact on the two dimensions of relationship quality, trust and commitment. This shows that when buyer and seller perceive that their partners will fulfill their commitments and have a strong sense of responsibility for this relationship, they will be more willing to maintain a longer-term and stable relationship with their partners, and then produce higher trust and commitment.

Secondly, the results show that trust and commitment have significant negative effects on opportunistic behavior, so the relationship quality between buyer and seller can reduce the occurrence of opportunistic behavior. When buyer and seller have a good cooperative relationship, it means that buyer and seller of both sides maintain close communication and exchange, which also avoids the increased cost caused by information asymmetry, improves the efficiency of cooperation, and makes buyer and seller of both sides make efforts to achieve longer-term goals and higher interests, so as to reduce the occurrence of opportunistic behavior.

Furthermore, this study found that trust and commitment in relationship quality play an intermediary role in the impact of opportunistic behavior. This is because psychological contract is subjective and does not require both parties to reach an agreement on perception, so psychological contract can only restrict their own attitude and behavior, and cannot directly control the behavior of others. Moreover, opportunistic behavior is a behavior that only focuses on “short-term interests,” and this “shortsightedness” largely comes from a sense of insecurity, that is, the partner cannot determine the stability of this cooperative relationship with the enterprise. However, this sense of insecurity and uncertainty often comes from the relationship management concept of “short-sightedness” and “one hammer deal” of the enterprise itself. This relationship concept will be reflected in the psychological contract, which will be reflected in the attitude and behavior of low trust and commitment, and then urge partners to choose self-interest opportunistic behavior to obtain short-term benefits. On the contrary, when a buyer and seller establish its own awareness of long-term cooperation, it will enhance its own psychological contract and believe the other party have a strong sense of responsibility for the commitments. In this way, there will be more trust, investment and closer communication between buyer and seller. Therefore, the occurrence of opportunistic behavior will be reduced and avoided.

Finally, the study found that the degree of dependence can positively regulate the impact of psychological contract on trust and commitment in relationship quality, that is, the higher the degree of dependence, the stronger the positive effect of psychological contract on trust and commitment. Therefore, when its partners have rare resources or high conversion costs, the enterprise's intention of long-term cooperation will be stronger. It also believes that the other party has the ability to bring benefits to and meet the needs of our party. Due to the scarcity of resources or the importance of the ability of its partners to our party, the enterprise is willing to take risks. At the same time, it also believes that it needs to perform its duties to promote the development of the relationship. This is reflected in the enhancement of trust and commitment of psychological contract to its partners.

### Theoretical Contributions and Managerial Implications

This article focuses on the dark side of channel relationship, selects enterprise opportunistic behavior as the research object, and further expands and explores on the basis of previous scholars' psychological contract and relationship quality, that is, opportunistic behavior, which has a certain contribution to the existing research results.

#### Theoretical Contributions

Firstly, this article enriches and expands the research of psychological contract in the field of relationship marketing. The existing research on psychological contract at home and abroad mainly focus on the relationship between employment (Kiazad et al., 2019), although some scholars have confirmed the existence of psychological contract in inter enterprise relationship through research (Kingshott, 2006; Gao et al., 2009; Blessley et al., 2018), but generally speaking, the research in this field is still in the exploratory stage, including the existing scales. In particular, the domestic research in this field is even less than that in foreign countries, with only a few articles. Therefore, this study further confirms the existence of psychological contract in relationship marketing under the background of Chinese environment, and has a significant impact on the quality of buyer-seller relationship.

Secondly, this article enriches the research on the dark side of relationship marketing. Over the years, many examples have proved that opportunistic behavior has great destructive power in enterprise cooperation, which will not only limit the value creation and performance level (Feng and Li, 2019), affect the enterprise's own reputation and reduce the innovation efficiency. It may also directly lead to the collapse of the cooperative relationship between the two sides. Scholars pay less attention to the dark side of channel relationship, mainly focusing on the positive impact of buyer-seller cooperation. Therefore, this article enriches domestic research in this field by studying the opportunistic behavior of buyer and seller in cooperative relationship.

In addition, this article tentatively applies psychological contract to the interpretation and governance of enterprise opportunistic behavior. Previous scholars have conducted extensive research and Discussion on the causes and results of enterprise opportunistic behavior (Hennart, 1988; Das and Rahman, 2010; Gould et al., 2016). This article connects the psychological contract with the opportunistic behavior of buyer and seller, and finds that the psychological contract produces a high degree of trust and commitment through the relationship quality, and then reduces the occurrence of opportunistic behavior, which fills the gap in the domestic research on the impact mechanism of psychological contract between buyer and seller on opportunistic behavior.

#### Managerial Implications

Based on this study on the psychological contract between buyer and seller, it confirms that the psychological contract exists in the relationship marketing between buyer and seller, and helps buyer and seller change the stereotype that the psychological contract only exists between employees and employers within the enterprise. It also helps buyer and seller realize the importance of psychological contract to the quality of bilateral cooperative relationship, so as to promote enterprise managers to further clarify the rights and obligations in the bilateral relationship.

Buyer and seller often restrict the behavior of both parties through formal contract terms, but opportunistic behavior often exists in the cooperation between buyer and seller. The exploration and discovery of this article can well-explain why the existence of formal contracts between buyer and seller cannot prevent the occurrence of selfish opportunistic behavior in transactions and cooperation. It is not enough for both buyer and seller to maintain a healthy cooperative relationship only by relying on a formal contract. It also needs to be considered from the psychological factors of the relationship, that is, the “sense of security” is very important. The research on psychological contract helps to improve the awareness of buyer and seller for their own and partners' opportunistic behavior.

This study also provides managerial implications for the maintenance and governance of buyer-seller relationship marketing. When establishing cooperative relationship with other buyer or seller, it is necessary to establish the awareness of long-term cooperation, which will strengthen the relationship quality of bilateral cooperation. This long-term orientation will be transmitted to their partners through communication or behavior expression, so as to increase each other's sense of security and reduce their opportunistic behavior. And if you want to enhance the trust and commitment of the other enterprise to us, you can strengthen the specific terms of its psychological contract. For example, give the other party what they need, give sufficient benefit feedback, etc.

### Research Limitation and Further Research

This article still has limitations and deficiencies, and has many problems and directions, which need to be continuously improved and expanded in future research. There are limitations in the depth and breadth of research. In the research on the influence path of psychological contract on enterprise opportunistic behavior, this article mainly considers the regulatory effect of dependence, but other factors, including special investment and supervision, will affect the occurrence of opportunistic behavior. Due to the limitation of time and energy, it cannot be explored in an all-round way. At the same time, in the research on relationship quality, this article only selects the intermediary role of trust and commitment, but there are still dimensions such as satisfaction, conflict, coordination and response to be explored. The data collection and measurement methods in this article also have limitations. The research mainly adopts the method of survey. Although the commonality deviation is avoided by filling in by two people, the data results are still subjective. Future research in this field can collect all-round information through interviews, observations and other ways.

## Data Availability Statement

The original contributions presented in the study are included in the article/supplementary material, further inquiries can be directed to the corresponding author/s.

## Author Contributions

LZ, YH, and LY contributed to conception and design of the study. QZ organized the database. LZ and YX performed the statistical analysis. LZ, QZ, and YX wrote the first draft of the manuscript. LZ, YH, LY, and QZ wrote sections of the manuscript. All authors contributed to manuscript revision, read, and approved the submitted version.

## Funding

This work was supported by Social Science Program of Beijing (Grant No. 18GLB040), Huiyuan Excellent Young Scholars Program of University of International Business and Economics (Grant No. 20YQ04), National Natural Science Foundation of China (Grant No. 71772013), National Natural Science Foundation of China (Grant No. 72171051), and the Fundamental Research Funds for the Central Universities of University of International Business and Economics (Grant No. CXTD11-04).

## Conflict of Interest

The authors declare that the research was conducted in the absence of any commercial or financial relationships that could be construed as a potential conflict of interest.

## Publisher's Note

All claims expressed in this article are solely those of the authors and do not necessarily represent those of their affiliated organizations, or those of the publisher, the editors and the reviewers. Any product that may be evaluated in this article, or claim that may be made by its manufacturer, is not guaranteed or endorsed by the publisher.
